# Spatiotemporal trends in neonatal, infant, and child mortality (1990–2019) based on Bayesian spatiotemporal modeling

**DOI:** 10.3389/fpubh.2023.996694

**Published:** 2023-02-09

**Authors:** Shaobin Wang, Zhoupeng Ren, Xianglong Liu

**Affiliations:** ^1^Institute of Geographic Sciences and Natural Resources Research, Chinese Academy of Sciences, Beijing, China; ^2^State Key Laboratory of Resources and Environmental Information System, Beijing, China

**Keywords:** spatiotemporal trends, neonatal mortality, Bayesian spatio-temporal modeling, health inequality, infant mortality, child mortality

## Abstract

**Background:**

Neonatal mortality rate (NMR), infant mortality rate (IMR), and child mortality rate (CMR) show a huge difference across countries, which has been posing challenges for public health policies and medical resource allocation.

**Methods:**

Bayesian spatiotemporal model is applied to assess the detailed spatiotemporal evolution of NMR, IMR, and CMR from a global perspective. Panel data from 185 countries from 1990 to 2019 are collected.

**Results:**

The continuously decreasing trend of NMR, IMR, and CMR indicated a great improvement in neonatal, infant, and child mortality worldwide. Further, huge differences in the NMR, IMR, and CMR still exist across countries. In addition, the gap of NMR, IMR, and CMR across the countries presented a widening trend from the perspective of dispersion degree and kernel densities. The spatiotemporal heterogeneities demonstrated that the decline degree among these three indicators could be observed as CMR > IMR > NMR. Countries such as Brazil, Sweden, Libya, Myanmar, Thailand, Uzbekistan, Greece, and Zimbabwe showed the highest values of b_1*i*_, indicating a weaker downward trend compared to the overall downward trend in the world.

**Conclusions:**

This study revealed the spatiotemporal patterns and trends in the levels and improvement of NMR, IMR, and CMR across countries. Further, NMR, IMR, and CMR show a continuously decreasing trend, but the differences in improvement degree present a widening trend across countries. This study provides further implications for policy in newborns, infants, and children's health to reduce health inequality worldwide.

## Introduction

The 2030 Agenda for Sustainable Development, issued by United Nations, provides a shared blueprint for peace and prosperity for humans on the planet, now and into the future. The Sustainable Development Goals (SDGs) are a global call to action to end poverty and protect the earth's environment and human health (1). Specifically, SDG 3 states that countries should aim to reduce neonatal mortality to at least 12 per 1,000 live births and under-five mortality to at least 25 per 1,000 live births by 2030. However, despite that progress, 5.2 million children died before reaching their fifth birthday in 2019, with almost half of those deaths, 2.4 million, occurring in the first month of life (2). Further, the mortality of newborns, infants (in the first year of life), and children (under 5 years old) shows a huge difference across countries. For example, in North America, the average neonatal mortality rate (NMR), infant mortality rate (IMR), and child mortality rate (CMR) in 2019 were relatively low, which were about 3.7, 5.4, and 6.3 deaths per 1,000 live births, respectively. In contrast, these indicators in developing countries in Sub-Saharan Africa present substantially higher levels at 27.5, 51.7, and 75.8 deaths per 1,000 live births in 2019, respectively, based on the United Nations Children's Fund data.

Accordingly, it is essential to investigate the spatiotemporal evolution of NMR, IMR, and CMR from a global perspective, which is the basis for developing health policies and facing the challenge of newborns, infants, and children's health. Some previous studies have investigated spatial differences or patterns of one or several countries, especially in the study of infant mortality. For instance, prior studies indicated that the progress of child survival had been uneven and high levels of child mortality persist in many countries, especially in Sub-Saharan African countries (3). Further, the global distribution of IMR was investigated and mapped at national and subnational levels (4). The regional pattern of IMR has been studied at the country level, such as in Brazil (5), Bangladesh (6), the U.S. (7), Austria (8), India (9), South Africa (10), Nepal (11), China (12) etc. In comparison, several studies on NMR and CMR have adopted a spatial approach at the regional level, such as in Africa (13) and Sub-Saharan Africa (14), and at the country or city level, such as in France (15), Malawi (16), Nigeria (17), etc.

Nevertheless, several gaps should be addressed. First, most previous studies have focused on NMR, IMR, and CMR separately, whereas few researchers have examined the evolution of the three indicators comprehensively. Thus, it may limit us from understanding the full picture of the world's most vulnerable population, i.e., newborns, infants, and children. Second, most of those prior studies were from a spatial or temporal perspective within one country. In contrast, the investigation across the country from a spatiotemporal perspective has been largely unexplored, which may hinder the evaluation of the health inequality of newborns, infants, and children on the global and regional scales. Third, it lacks studies on health inequality in newborns, infants, and children's health improvement, especially from the cross-country perspective. So, it may be inconducive to make policy priority for intervention in newborns, infants, and children's health to reduce health inequality in the global community.

Accordingly, to fill the gaps mentioned above, we examine the spatiotemporal trends in NMR, IMR, and CMR at the global, regional, and national levels. This study is the first to investigate the global spatiotemporal variation of the three indicators under one framework. To achieve these aims, we use a Bayesian spatiotemporal model to explore the spatiotemporal trends. Panel data on NMR, IMR, and CMR are collected in 185 countries from 1990 to 2019. This paper could provide the following contributions. First, this paper can offer a global perspective on a spatiotemporal basis of the comprehensive assessment of the changes in child mortality in under-5, infant, and neonatal age groups in the world, different from the traditional study. Second, the results generated by this paper can help policymakers assess the relative state and shed light on guiding priority setting for optimal medical resource allocation by the public health administration and donors on newborn, infant, and children's health on a global scale.

## Methods

### Data sources and indicators

According to the global measurements of healthcare performance, neonatal, infant, and child mortality rates (NMR, IMR, and CMR) are the most predominant indicators of newborns', infants', and children's health conditions. Therefore, this study collected these three indicators as the core health outcomes as follows. 1) Neonatal mortality rate (per 1,000 live births) is the number of neonates dying before reaching 28 days of age per 1,000 live births in a given year. 2) Infant mortality rate (per 1,000 live births) is the number of infants dying before reaching 1 year of age per 1,000 live births in a given year. 3) Child mortality rate (under-five mortality rate) is the probability per 1,000 that a newborn baby will die before reaching age five if subject to age-specific mortality rates of the specified year.

In the current paper, the data on the mortality rates of neonatal, infant, and children are from the new round of estimates developed by the United Nations Inter-agency Group for Child Mortality Estimation (UN-IGME), which is led by the United Nations Children's Fund (UNICEF) and includes the World Health Organization (WHO), the World Bank Group and the United Nations Population Division of the Department of Economic and Social Affairs as full members (2). Further, most countries do not have well-functioning vital registration systems in the developing world. Therefore, household surveys, such as the UNICEF-supported Multiple Indicator Cluster Surveys and the USAID-supported Demographic and Health Surveys, and periodic population censuses have become the primary sources of data on childhood mortality in developing countries. Finally, given the data availability, a total of 185 countries with NMR, IMR, and CMR from 1990 to 2019 were collected to explore spatiotemporal trends. In this study, the data for any sample missing one or more data elements were thrown out, and the countries with missing data were shown on the maps.

### Bayesian spatiotemporal model

The Bayesian spatiotemporal model is widely used to explore the spatiotemporal trends in health metrics such as life expectancy (18), mortality (19), and disease prevalence (e.g., diabetes) (20). In this study, we used a Bayesian spatiotemporal model with a Gaussian distribution that can explicitly characterize each country's spatial pattern, temporal trend, and local trends. This model uses a similar specification to follow the formula proposed by a prior study (21), and has been used to model life expectancy (18).

Let *y*_*it*_ denotes the NMR, IMR or CMR in year *t* (*t* = 1990, 1991, …, 2019) of the *i* th (*i* = 1, 2, ..., 185) country. The ln*y*_*it*_ was assumed follow a normal distribution and can be modeled as ln*y*_*it*_ ~ *Normal* (μ_*it*_, σ^2^). The μ_*it*_ can be modeled using the following formula:


(1) $$\begin{array}{l} \mu_{it}=\;\alpha +s_{i}+\;b_{0}t+\;v_{t}+\;b_{1i}t+\;\varepsilon_{it} \end{array}$$

In Equation (1), μ_*it*_ denotes the expected value of ln*y*_*it*_ in country *i* and year *t*; σ^2^ is the corresponding variance of ln*y*_*it*_; α is the intercept which represents overall ln*y*_*it*_ over the years from 1990 to 2019; The *s*_*i*_ indicates the spatial random effect, which can be formulated as *s*_*i*_ =*u*_*i*_ + υ_*i*_. *u*_*i*_ and υ_*i*_ are structured and unstructured random effects, respectively. The *s*_*i*_ describes a “stable” spatial pattern of ln*y*_*it*_ across the study period 1990–2019, that is helpful to identify the countries with high and low NMR, IMR or CMR. If *s*_*i*_ > 0, indicating the ln*y*_*it*_ in country *i* is higher than global average value of ln*y*_*it*_ across the whole study period. *b*_0_*t* + *v*_*t*_ indicates the global temporal trend common to all 185 countries. *b*_0_*t* measures the linear trend and *v*_*t*_ measures the nonlinear temporal effect. *b*_1*i*_*t* is used to measure local temporal trends of each country. The term *b*_1*i*_ measures the country-specific change rate in ln*y*_*it*_ over time, which describes the departure from *b*_0_ for each country. For example, if *b*_0_ > 0 and *b*_1*i*_ > 0, indicating NMR, IMR or CMR increased over time, and *ith* country increased faster than the global increase rate. ε_*it*_ is a Gaussian noise error.

For the prior distributions to the random effect *s*_*i*_ and *b*_1*i*_, we assign the Besag York Mollie (BYM) model (22). The BYM model is a convolution of *u*_*i*_ and υ_*i*_, where *u*_*i*_ is modeled as the intrinsic conditional autoregressive (iCAR) prior with a spatial adjacency matrix ***W*** to incorporate spatial autocorrelation in parameters *u*_*i*_. In this study, the ***W*** of size 185×185 is defined by first-order queen contiguity. Where *w*_*ij*_ = 1 if country *i* and *j* share a border and *w*_*ij*_ = 0 otherwise. The temporal noise *v*_*t*_ is modeled $v_{t}\sim N\left(0,\sigma_{v}^{2}\right)$. The term ε_*it*_ is assumed as $\varepsilon_{it}\sim N\left(0,\sigma_{\varepsilon }^{2}\right)$. Follow by previous studies, a noninformative prior N(0,1000) is assigned to α, *b*_0_. A positive half-normal priors with means of zero and variances of 10 are assigned to all random effect standard deviations (e.g., σ_*v*_ and σ_ε_) (21, 23).

Bayesian spatiotemporal model was implemented by using OpenBUGS software. We ran two Markov Chain Monte Carlo (MCMC) chains for each model with 200,000 iterations. The first 180,000 iterations were removed as burn-in. The posterior distributions of model parameters were obtained on MCMC samples based on every 10th iteration for the remaining 20,000 MCMC iterations with two chains. We used the Brooks-Gelman-Rubin (BGR) ratio (24) to evaluate the convergence diagnosis. The closer the ratio is to 1.0, the better the model convergence is (21). Only 6.9% of parameters had a BGR ratio >1.05.

### Distribution pattern test

Kernel density estimation (KDE) was widely applied to estimate the density function based on a set of random variables without any hypothesis on the data distribution. In this study, the general difference in NMR, IMR, and CMR was depicted based on KDE, which can be calculated as follows (25):


(2) $$\begin{array}{l} \hat{f}\left(x\right)=\frac{1}{n}\overset{t}{\underset{i=1}{\sum }}K\left(\frac{x_{i}-x}{h}\right) \end{array}$$

In Equation (2), *n* is the total number of spatial units (countries in this study); x_*i*_ represents the value of NMR, IMR, and CMR in *i*th country. *h* is the bandwidth. *K* is a kernel density function by using the Epanechnikov kernel method.

The coefficient of variation (CV) is calculated to measure whether the degree of dispersion of NMR, IMR, and CMR across countries decreases or not during the study period as follows:


(3) $$\begin{array}{l} CV_{i,t}=\frac{\sqrt{\frac{1}{n}\sum_{i=1}^{n}{\left(x_{i,t}-{\bar{x}}_{i,t}\right)}^{2}}}{{\bar{x}}_{i,t}} \end{array}$$

In Equation (3), *i* and *t* refer to the country and year, respectively. *n* denotes the total number of countries. *x* represents NMR, IMR, and CMR. $\bar{x}$ is the mean value of NMR, IMR, and CMR in *i*th country in year *t*. σ_*i, t*_ represents the standard deviation of NMR, IMR, and CMR.

### Regional division

**Figure 1:**
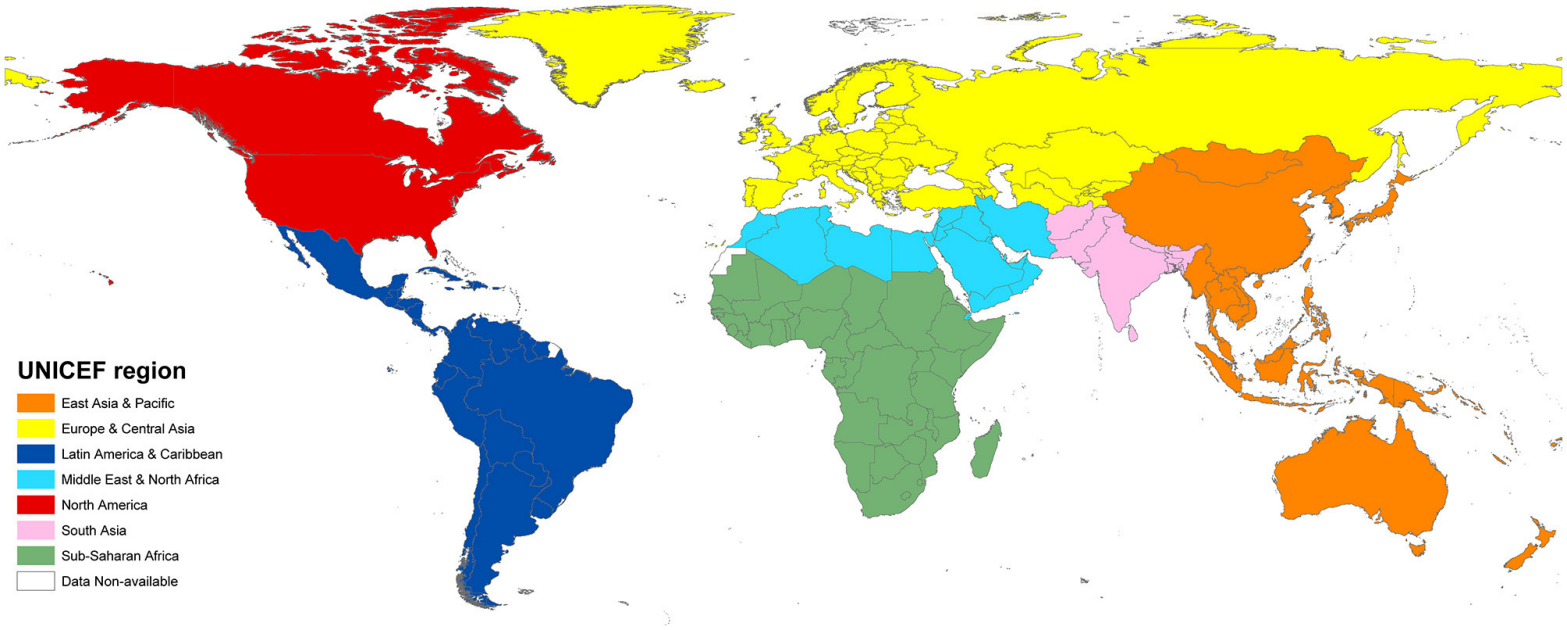
Region division by UNICEF.

Severn regions in the world are divided based on the classifications of UNICEF (https://data.unicef.org/regionalclassifications/), which include East Asia and Pacific, Europe and Central Asia, Latin America and the Caribbean, Middle East and North Africa, North America, South Asia, Sub-Saharan Africa (Figure 1). The spatial distribution maps in this paper are drawn by using ArcGIS (version 10.2).

## Results

### Global variations

**Figure 2:**
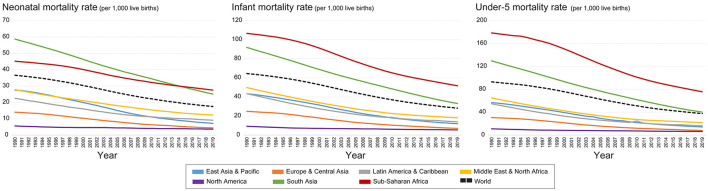
Change of NMR, IMR, and CMR in the world and different regions by UNICEF from 1990 to 2019.

The temporal trends in NMR, IMR, and CMR at the global average level and in seven regions divided by UNICEF were depicted from 1990 to 2019 (Figure 2). Several points can be demonstrated. First, a general decreasing trend in each region and the global average level can be seen during the study period, especially for the regions of South Asia and Sub-Saharan Africa, which have a substantial decrease in the three indicators. Second, great differences among regions can be observed as well. For example, the NMR, IMR, and CMR in South Asia and Sub-Saharan Africa are still higher than the world average levels and are evidently higher than those in North America and Europe-Central Asia, which have the lowest values in the world.

**Figure 3:**
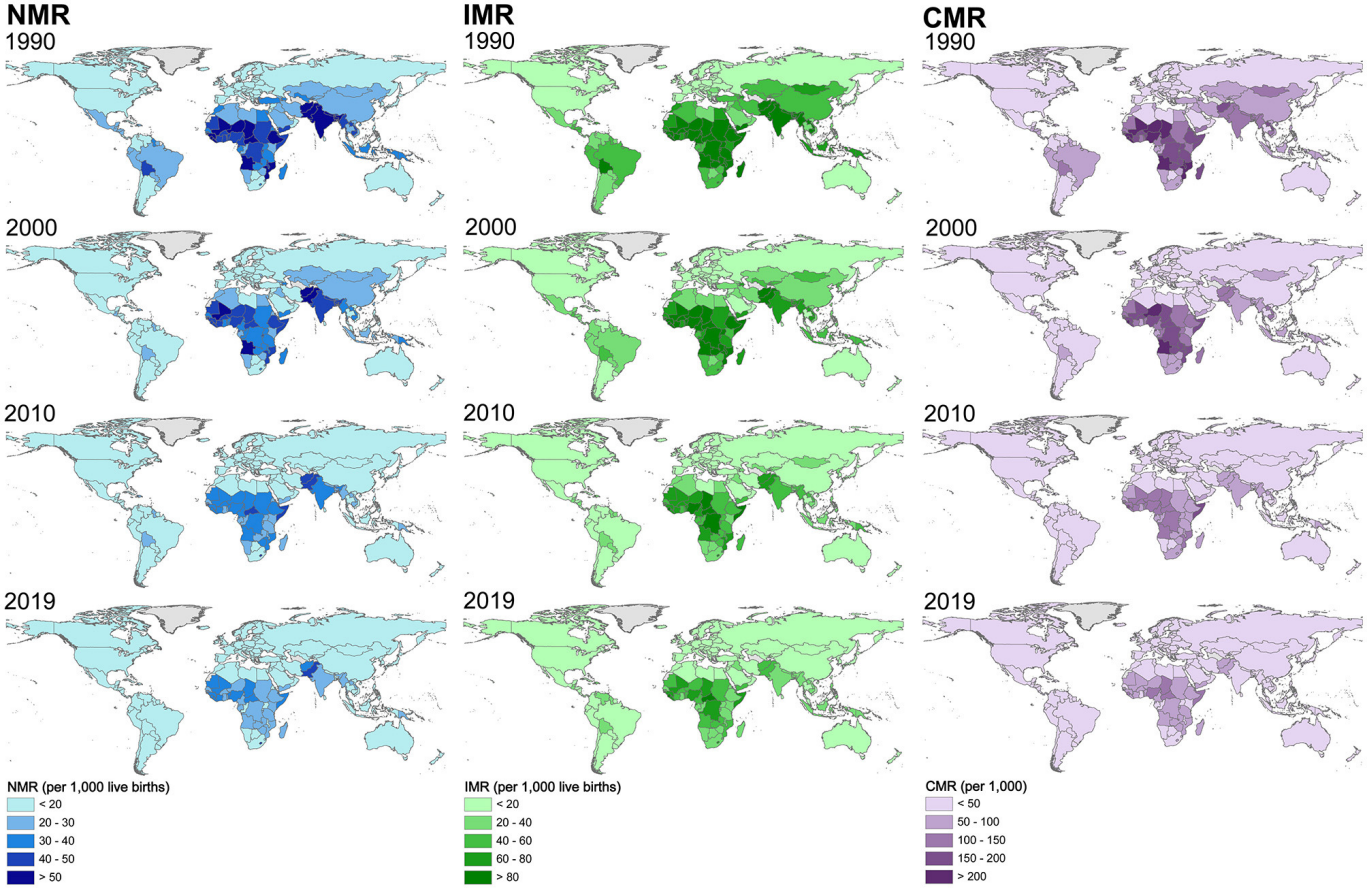
Spatial distribution of NMR, IMR, and CMR in each country in the world from 1990 to 2019 with a 10-year interval.

The global distribution of the three indicators (NMR, IMR, and CMR) in each country from 1990 to 2019 were illustrated (Figure 3). Two points can be concluded from the variation trend. First, in general, the decrease in the three indicators can be observed in the world during the study period, especially in developing countries in Africa, Latin America, and Asia. The decreasing trend indicated a great global improvement in infant and child health since 1990. Second, the evident spatial differences of the three indicators still exist across countries, though a great improvement has been obtained. For example, some countries in Sub-Saharan Africa, South Asia, and Southeast Asia still had higher levels of NMR, IMR, and CMR in 2019 (Figure 3). In comparison, countries in America, Europe, East Asia, and Oceania show lower levels of these three indicators.

**Figure 4:**
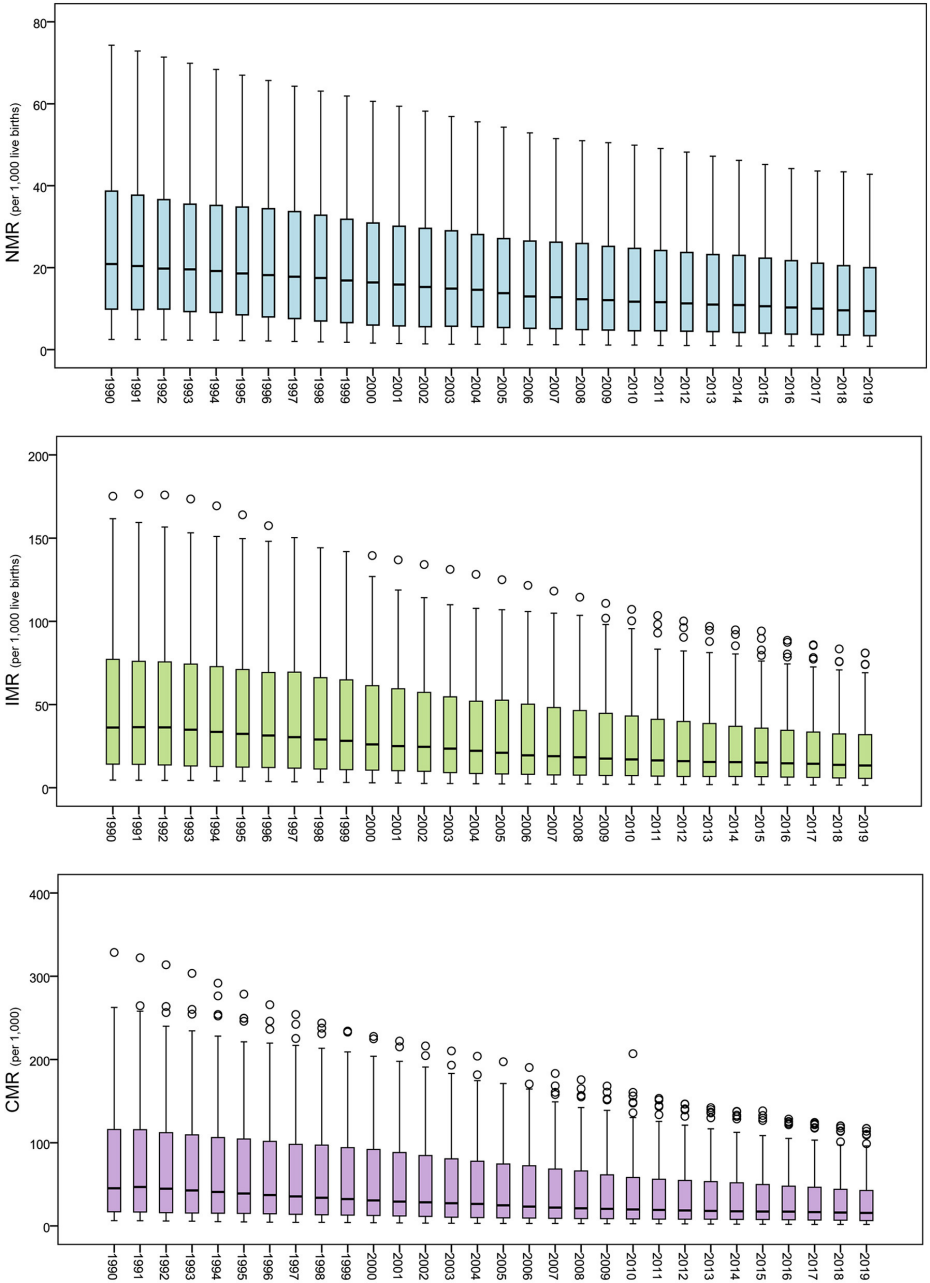
Boxplot of the global NMR, IMR, and CMR from 1990 to 2019.

Boxplots of the global NMR, IMR, and CMR were depicted from 1990 to 2019 (Figure 4). It can be seen that the distribution ranges of the global NMR, IMR, and CMR decreased, with the difference in the indicators across countries and regions continuously decreasing during the study period.

**Figure 5:**
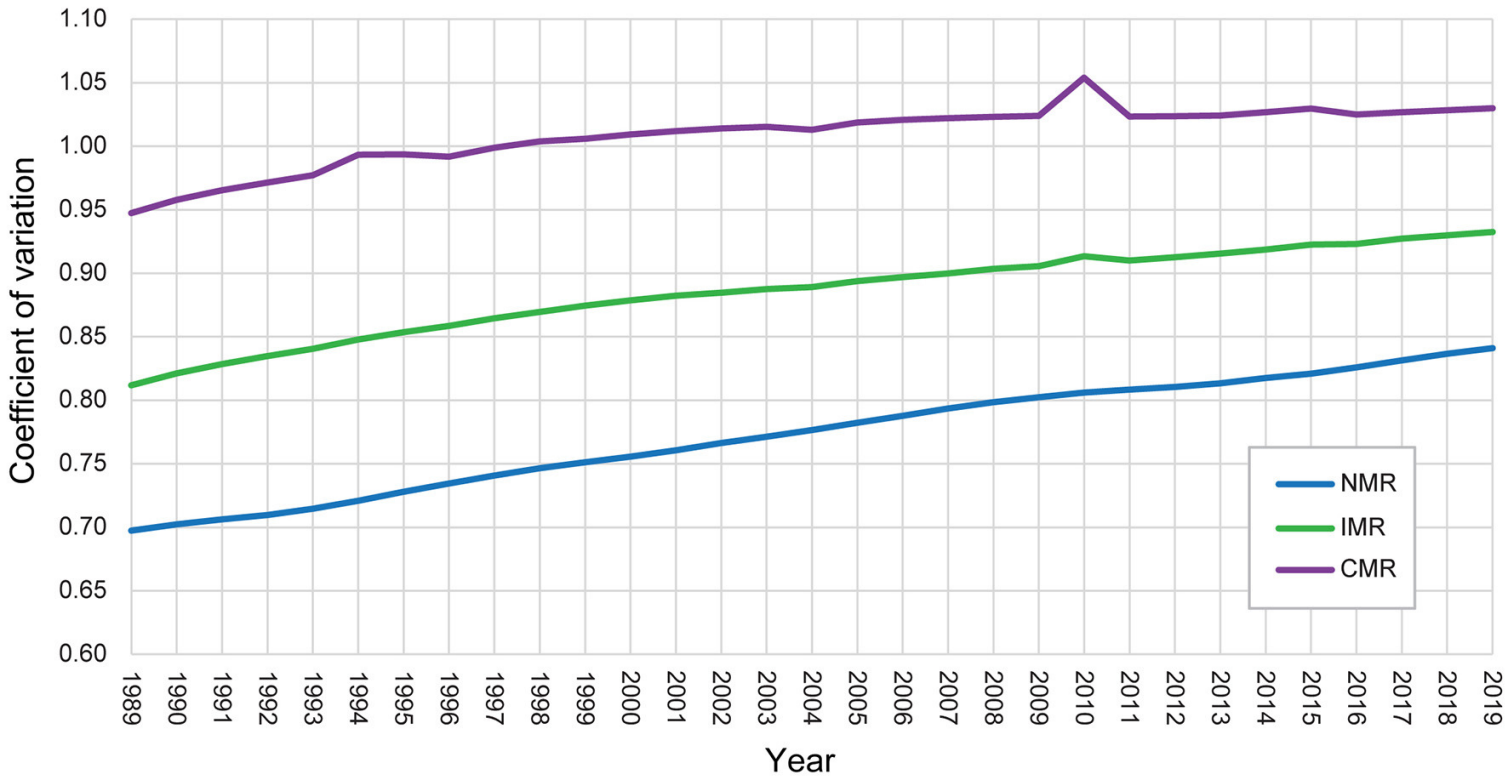
Trend of the coefficient of variation of global NMR, IMR, and CMR from 1990 to 2019.

The CV of global NMR, IMR, and CMR from 19906 to 2019 is illustrated in Figure 5, showing an increasing trend. Hence, the increasing trend of the dispersion degree of the three indicators can be found, though the distribution ranges show a declining trend.

**Figure 6:**
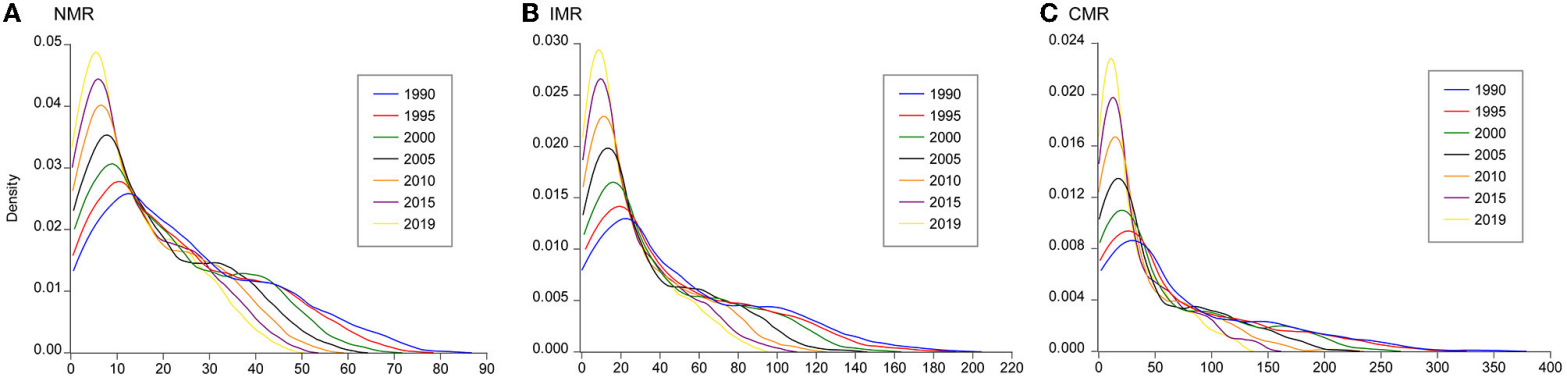
Kernel density estimations of global **(A)** NMR, **(B)** IMR, and **(C)** CMR from 1990 to 2019 with a five-year interval.

In addition, the estimated kernel densities of global NMR, IMR, and CMR can provide further evidence of the evolution trend (Figure 6). First, the Kernel density curves of global NMR, IMR, and CMR exhibited right-skewed distributions with an upward trend of kurtosis values during the study period. Second, the distribution area of the Kernel density curves moved left and became smaller over time. Third, the three indicators showed similar temporal variation characteristics with the kurtosis values as NMR > IMR > CMR.

In sum, several features of the variation of global NMR, IMR, and CMR can be obtained based on the analysis in this section. First, global NMR, IMR, and CMR exhibited an evident decline trend from 1990 to 2019, indicating a great improvement in neonatal, infant, and child mortality in the world. Second, huge differences in the NMR, IMR, and CMR still exist across countries. Some countries in Sub-Saharan Africa, South Asia, and Southeast Asia still have higher neonatal, infant, and child mortality levels. Third, the gap of NMR, IMR, and CMR across countries is widening from the perspective of the three indicators' dispersion degree and kernel densities.

### Common spatial patterns

**Figure 7:**
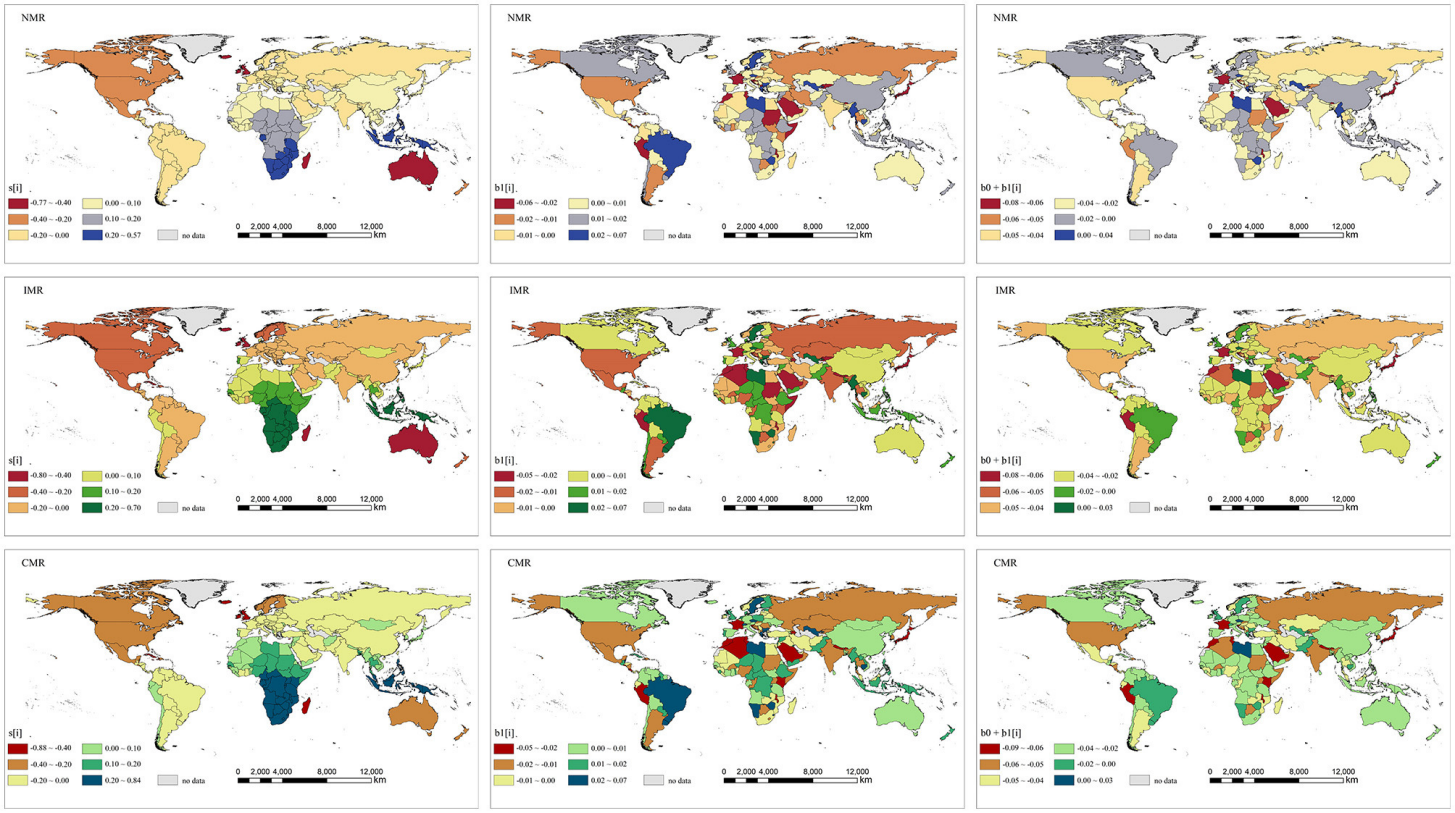
Maps of the spatial relative departures, local temporal departures and local temporal trends of NMR, IMR, and CMR in 185 countries, 1990–2019. **(Left column)** The common spatial pattern (posterior medians of the spatial relative departures s_i_), **(Middle column)** the local departures from the overall temporal trend (the posterior medians of local temporal departures b_1i_), **(Right column)** the country-specific temporal trends (the posterior medians of b_0_+b_1*i*_).

The estimated common spatial pattern of NMR, IMR, and CMR is illustrated in Figure 7. The spatial distribution of s_i_ describes a “stable” spatial pattern of NMR, IMR, or CMR across the study period from 1990 to 2019, which helps identify the countries with high and low NMR, IMR, or CMR. It shows similar spatial patterns of global NMR, IMR, and CMR. The countries shaded in red and orange are mainly located in North America, Europe, Australia, and New Zealand, with the lowest values of s_i_, indicating that NMR, IMR, and CMR in this region are much lower than the overall level. These countries mentioned above are consistent with the distribution of developed countries. In contrast, the countries colored in green or blue, which are mainly in Africa and South-East Asia, exhibit higher values, indicating a relatively higher level compared to the global overall average level. Other regions in the world, e.g., East Asia, Russia, the Middle East, and South America, are in yellow, indicating a similar level compared with the world's overall level of NMR, IMR, and CMR. The results suggest that the common spatial pattern of NMR, IMR, and CMR during the study period has an obvious spatial disparity (Figure 7).

### Overall and local temporal trends

**Figure 8:**
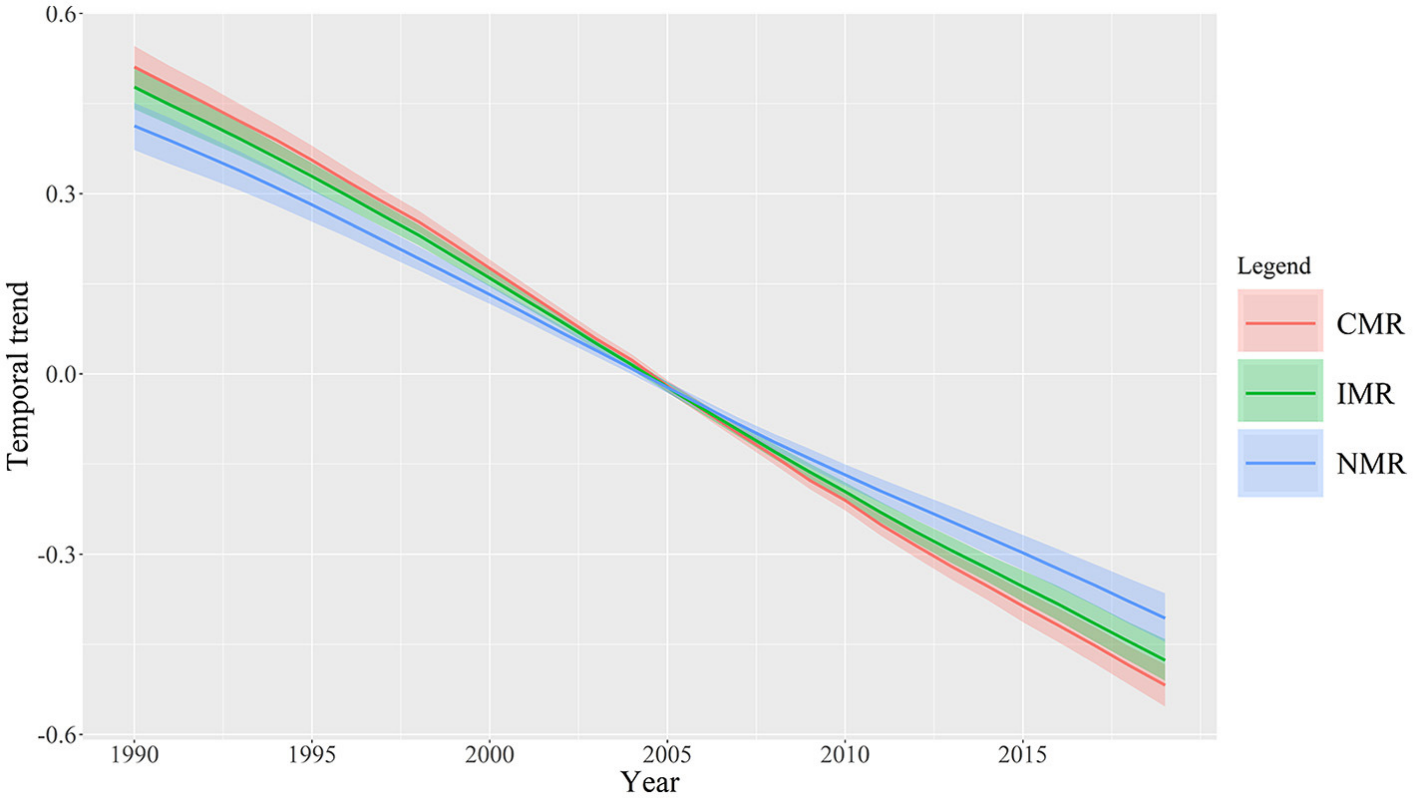
Overall temporal trends of NMR, IMR, and CMR with 95% CI (the posterior medians of b_0_t+v_t_) during the study period 1990–2019.

The overall temporal trend of NMR, IMR, and CMR (b_0_*t* + *v*_t_) were estimated based on the Bayesian spatiotemporal model, which exhibited a general decrease trend from 1990 to 2019 (Figure 8). The posterior median of b_0_ is −0.03398, −0.03404, and −0.03679, which indicates global NMR, IMR, and CMR are decreasing by 3.34, 3.35, and 3.61% per year, respectively. Hence, the three metrics decreased at different rates, and it can be concluded that the CMR decreased faster than IMR and NMR over the study period.

To extract the local temporal trends of NMR, IMR, and CMR, we estimated the country-specific temporal trends (b_0_ + b_1i_) and local departures from the overall temporal trend (b_1i_). We observed that there were huge differences in country-specific temporal trends of NMR, IMR, and CMR across countries in the world (Figure 7). For NMR, only several countries, such as Libya, Myanmar, Uzbekistan, Greece, and Zimbabwe, showed an increasing trend during the study period. For IMR and CMR, only Libya and the Czech Republic showed increasing trends. Other countries and regions presented decreased trends in NMR, IMR, and CMR. In specific, the countries such as France, Japan, Saudi Arabia, Peru, and Morocco showed red or dark orange color, indicating the faster-decreased rate of the NMR, IMR, and CMR during the study period. Other countries in America, Asia, Europe, and Oceania showed slower decreased trends in the NMR, IMR, and CMR.

The local departures from the overall temporal trend, quantified by the parameter b_1*i*_, is shown in Figure 7. The overall temporal trend exhibited a downward movement; thus, the local departures from the overall temporal trend of NMR, IMR, and CMR in the *i*th country have a significant downward trend than the overall downward trend if the parameter b_1*i*_ is lower than 0 and has a weaker downward trend than the overall downward trend if b_1*i*_ is higher than 0. It is clear that there were obvious differences in the spatial distribution of the local departures from the overall temporal trend of each country (b_1*i*_) compared with the overall temporal trend during the study period 1990–2019 (Figure 7). Specifically, countries such as France, Japan, Saudi Arabia, Peru, Nepal, Morocco, and Algeria showed the lowest values of b_1*i*_, indicating that these countries made huge progress in decreasing NMR, IMR, and CMR compared with other countries in the world. On the other hand, the highest values of b_1*i*_ in countries such as Brazil, Sweden, Libya, Myanmar, Thailand, Uzbekistan, Greece, and Zimbabwe, suggested that these countries decreased NMR, IMR, and CMR at a relatively slower rate compared to the overall decreased trend in the world.

## Discussion

The spatial distribution and variation of newborns, infants, and children's mortality worldwide have been largely unexplored. The current study is one of the first papers that exhibited the spatial difference in NMR, IMR, and CMR from a global perspective. Further, the spatiotemporal distribution and variation can be accessed at the overall and local level from the space-time coupling process in the current study, which could extensively reveal the spatiotemporal heterogeneities in NMR, IMR, and CMR globally. In specific, several key points can be discussed as follows.

In prior papers, the spatial distribution and variation trends of NMR, IMR, and CMR have been conducted at the cross-country level. For example, a Bayesian geostatistical analytical framework estimated child and neonatal mortality across 46 countries in Africa from 2000 to 2015 (13). Similarly, the sub-national and regional spatial distribution of CMR were depicted from 1990 to 2015, indicating considerable heterogeneity across Africa (26). Meanwhile, the spatial distribution of IMR was investigated for 192 countries from 1990 to 2011, which indicated significant spatial clustering of high-surrounded confined to Sub-Saharan Africa with some countries in Central Asia, the Middle East, and Cuba, and low- surrounded confined to Europe and the U.S. (27). Compared with prior studies, our findings revealed a comprehensive picture of the world's variation of NMR, IMR, and CMR from 1990 to 2019. Further, huge differences in the NMR, IMR, and CMR can be seen across countries. Some countries in Sub-Saharan Africa, South Asia, and Southeast Asia still have higher neonatal, infant, and child mortality levels. For the first time, the current study investigated the spatiotemporal features of NMR, IMR, and CMR at the country level in the world, which can offer new evidence about infant and child health using Bayesian spatiotemporal modeling. Different from previous studies, our results disassemble the overall spatial distribution and variation, overall temporal trend, and local trend from the complex space-time coupling process to extensively investigate the spatiotemporal heterogeneities in NMR, IMR, and CMR globally. In specific, countries such as Brazil, Sweden, Libya, Myanmar, Thailand, Uzbekistan, Greece, and Zimbabwe showed the highest values of b_1*i*_, indicating the weaker downward trend compared to the overall downward trend in the world.

Moreover, our findings show that the distribution of s_i_ which presents an apparent zonal distribution and spatial clustering. In contrast, the patterns of b_1*i*_ and b_0_ + b_1*i*_ exhibit a discrete distribution in space. These findings indicate that the levels and improvement degree of NMR, IMR, and CMR show different distribution patterns across countries. s_i_ shows the zonal distribution and spatial clustering, and the patterns of b_1*i*_ and b_0_ + b_1*i*_ shows the discrete spatial distribution. Accordingly, our findings could help to further understand the global trends in newborns, infants, and children's health improvement. On the one hand, the declining trend of global NMR, IMR, and CMR indicated great improvement in neonatal, infant, and child mortality worldwide. This finding is in line with previous studies that revealed that substantial progress had been accomplished in reducing child mortality across countries worldwide (28, 29). On the other hand, huge differences in the NMR, IMR, and CMR can be observed across countries. Their gaps widen from the perspective of dispersion degree and kernel densities of the three indicators. Specifically, we identified the countries with a weaker downward trend compared to the overall global downward trend of NMR, IMR, and CMR, such as Brazil, Sweden, Libya, Myanmar, Thailand, Uzbekistan, Greece, and Zimbabwe. Similarly, it has been found that the decline of NMR has been slowest in the regions with high NMR (30). Based on our findings, the differentiation phenomenon of global NMR, IMR, and CMR is becoming increasingly severe from 1990 to 2019. Overall, the enlarging gap of global NMR, IMR, and CMR may be due to the slower improvement in Sub-Saharan Africa, Asia, and South America than the overall global levels.

Furthermore, our findings could further explain the differences between NMR, IMR, and CMR. For example, mortality in the neonatal period tends to decline more slowly than in the post-neonatal period (1–59 months) (31). Similarly, accelerated, faster-than-anticipated reductions in CMR occurred in many lower-income countries, but the decrease in NMR was mainly concentrated in higher-income countries (28). In the current study, CMR exhibited the fastest decline and followed by IMR and NMR, which may indicate the different influencing factors of the three indicators. Some studies indicated that infectious diseases and nutritional deficiencies dominate CMR, especially in low-income countries (28, 32). This trend reflected the epidemiological transitions and further shed light on the effective public health strategies for reducing CMR. In comparison, NMR accounts for almost 40% of CMR globally, which could be associated with a complex chain of factors including but not limited to socioeconomic, biological, and healthcare-related factors (31). The NMR remains high in Sub-Saharan countries, and two-thirds of countries at risk of missing the SDG neonatal mortality target are in Sub-Saharan Africa (33). The current paper revealed that the decline degree among these three indicators could be observed as CMR > IMR > NMR during the study period, indicating the inequality challenge in improving newborns, infants, and children's health. In policy scenario, improvement in NMR, especially in low-income countries, may face more severe challenges than in high-income countries.

This study could provide extra evidence and implications for inequality challenges in improving newborns, infants, and children's health. In general, health inequality in newborns, infants, and children's health improvement should be paid more attention to by the global community. Our findings reveal the difference between levels and improvement degree of NMR, IMR, and CMR in space, which may provide further evidence of the enlarging gaps of these three indicators worldwide. Notably, several countries with high levels of b_1*i*_ and b_0_ + b_1*i*_, such as Brazil, Sweden, Libya, Myanmar, Thailand, Uzbekistan, Greece, and Zimbabwe, are detected at a relatively slower rate compared to the overall decreased trend of NMR, IMR, and CMR in the world. Thus, this finding may offer some implications for the policy priority for intervention in newborns, infants, and children's health to reduce health inequality globally.

Nevertheless, our analysis is not without limitations. First, the mechanisms of the space-time process and the influencing factors of NMR, IMR, and CMR need further study. For instance, we found that several countries show relatively slower rates of NMR, IMR, and CMR compared to the overall decreased trend globally. Nevertheless, these countries (e.g., Brazil, Sweden, Libya, Myanmar, Thailand, Uzbekistan, Greece, and Zimbabwe) showed various levels of socio-economic development. Therefore, given the SDGs and the highly heterogeneous trends in the spatiotemporal variation of global NMR, IMR, and CMR, it is crucial to comprehensively assess factors that affected mortality trends in the past and identify which ones might further improve newborn, infant, and child survival in the future. Second, although the quantity of data available to estimate NMR, IMR, and CMR is relatively high for most countries, the potential for bias exists across the various data sources, especially in the countries with the highest stillbirth burdens (34). Third, our findings of spatiotemporal distribution and variation in this study are based on historical data. In the future, spatiotemporal patterns can be predicted to evaluate the trends and whether the country or region would meet SDGs for reducing newborn, infant, and child mortality.

## Conclusions

This paper has demonstrated the spatiotemporal distribution patterns and trends in NMR, IMR, and CMR globally. The following conclusions can be drawn from this study:

1. The results showed a continuously decreasing trend in NMR, IMR, and CMR from 1990 to 2019, indicating a great improvement in neonatal, infant, and child mortality worldwide. Further, huge differences in the NMR, IMR, and CMR still exist across countries. Some countries in Sub-Saharan Africa, South Asia, and Southeast Asia still have higher neonatal, infant, and child mortality levels. In addition, the gap of NMR, IMR, and CMR across countries is widening from the perspective of the three indicators' dispersion degree and kernel densities.
2. This study investigated the global spatiotemporal heterogeneities in NMR, IMR, and CMR by using Bayesian spatiotemporal modeling. First, NMR, IMR, and CMR showed similar spatial patterns of the spatial components during the study period. Second, the decline degree among these three indicators can be observed as CMR > IMR > NMR. Third, countries such as Brazil, Sweden, Libya, Myanmar, Thailand, Uzbekistan, Greece, and Zimbabwe showed the highest values of b_1*i*_, indicating a weaker downward trend compared to the overall downward trend in the world. Furthermore, our findings revealed that the decline degree among these three indicators indicated the difference in the improvement of newborns, infants, and children health improvement. CMR presented the fastest decline and followed by IMR and NMR.
3. Our findings could provide further policy implications. The global community should pay more attention to health inequality in newborns, infants, and children. In the actual scenario, the inequality in health improvement challenges indicates NMR > IMR > CMR, especially in low-income countries in the future. Several countries are detected at a relatively slower rate compared to the overall decreased trend of NMR, IMR, and CMR worldwide. In sum, this paper offers new implications for the policy priority for intervention in newborns, infants, and children's health to reduce health inequality worldwide.

## Data availability statement

The original contributions presented in the study are included in the article/supplementary material, further inquiries can be directed to the corresponding author.

## Author contributions

SW and ZR designed the study, wrote the first draft of the manuscript, and reviewed and edited the document. SW acquired the data. XL and ZR analyzed the data. ZR acquired the funding for this project. All authors interpreted the data, read, revised, and approved the final manuscript.
